# Retroperitoneal lymphatic malformation in a dog

**DOI:** 10.1186/s13028-020-0506-9

**Published:** 2020-02-01

**Authors:** Floor Driessen, Tim Cushing, Stephen John Baines

**Affiliations:** 1 Department of Clinical Sciences of Companion Animals, Faculty of Veterinary Medicine, Utrecht University, Yalelaan 108, 3584 CM Utrecht, The Netherlands; 2 Cytopath Veterinary Pathology, PO Box 24, Ledbury, Herefordshire HR8 2YD UK; 3 Department of Soft Tissue Surgery, Willows Veterinary Centre and Referral Service, Highlands Road, Shirley, Solihull, West-Midlands B90 4NH UK

**Keywords:** Bladder, Canine, Computed tomography, Cyst, Histopathology, Immunohistochemistry, LYVE-1, PROX-1

## Abstract

**Background:**

Lymphatic vascular malformations are rare findings in canine patients with six reports available in veterinary literature. Retroperitoneal cystic lymphatic malformations have not been described previously in canine patients and neither has the use of immunohistochemistry to determine their origin, i.e. vascular versus lymphatic.

**Case presentation:**

An 8-year-old neutered female Cocker spaniel was referred for pollakiuria, dysuria and a painful abdomen. Computed tomography scanning of the abdomen showed a fluid filled structure adjacent to the urinary bladder. During surgical exploration, a thin walled cystic structure with sero-haemorrhagic fluid was found, extending from the retroperitoneal space into the abdomen. The mass was excised and submitted for histopathology, revealing a cystic mass lined by a fibrovascular capsule within the retroperitoneal/mesenteric adipose tissue. The inner surface of the cyst was lined by a single layer of bland, flattened spindle cells. Intramural blood vessels were well differentiated, with perivascular haemorrhage. On recurrence 11 months later, the mass was excised for the second time and a PleuralPort (Norfolk Animal products) was placed. Fifteen months after initial presentation, progression occurred with haemorrhagic fluid in the cystic space, pleural- and abdominal cavities and the owners opted for euthanasia. Histopathology and positive immunohistochemistry for lymphatic markers lymphatic vessel endothelial hyaluronic acid receptor-1 (LYVE-1) and prospero homeobox protein-1 (PROX-1) confirmed a lymphatic vascular origin of the cystic structure.

**Conclusions:**

To our experience, a definitive diagnosis of retroperitoneal cystic malformation of lymphatic origin could be done only by combining the clinical presentation, advanced imaging, histopathology and LYVE-1 and PROX-1 immunohistochemistry. This is the first report of a vascular malformation in a dog where immunohistochemistry was used to make a final diagnosis. A lymphatic malformation, even if rare, should be added on the list of the differential diagnosis in a patient with a retroperitoneal cystic structure containing serohaemorrhagic fluid. Results of this case report can aid in diagnosis of future cases, however, further studies on therapy and management are needed to provide additional information about optimal treatment of these patients.

## Background

Vascular anomalies can occur due to tumoral cell proliferation or abnormal development of vascular endothelium (malformations) [1]. Congenital malformations with a lymphatic origin are uncommon in veterinary literature and have traditionally been grouped under the term lymphangioma, suggesting a neoplastic process, but since they are regarded as an abnormality of morphogenesis these are not true neoplasms [2, 3]. In human literature lymphangiomas are renamed to lymphatic malformations. However, both classifications are still used concurrently and synonymously, depending on the Society involved (World Health Organization (WHO)/International Society for the Study of Vascular Anomalies). As both classifications are still used interchangeably, review of the literature can be confusing and making differentiation between malformative and neoplastic lesions difficult [3, 4].

A review of the veterinary literature suggests that the vast majority of vascular malformations are of blood vessel origin [5–15]. Only few reports confirm a lymphatic vessel origin of vascular malformations in the skin, intestines, liver and mammary gland [16–21]. Veterinary reports that manage to make a suggestion for a lymphatic origin often refer to a neoplastic origin to the lesion [16–18]. The discrepancy in number of reported cases between vascular malformations compared to lymphatic malformations, likely stems from a lack of diagnostic modalities to adequately differentiate between vascular- and lymphatic endothelium. Halsey et al. [22] and Galeotti et al. [23] describe the successful use of lymphatic vessel endothelial hyaluronic acid receptor-1 (LYVE-1) and prospero homeobox protein-1 (PROX-1) immunohistochemical stains to differentiate between lymphatic over vascular origin in cutaneous angiosarcomas in 20 dogs and one cat. These stains have not been used so far for differentiation of vascular malformations in dogs and cats, compared to human medicine, where these stains have proven to successfully differentiate between lymphatic- and blood vessel origin of vascular malformations [23–28]. This report describes an unusual cystic lymphatic lesion in the retroperitoneal space in a dog.

## Case presentation

An 8-year-old neutered female Cocker spaniel was presented to the referring veterinary surgeon with a 24 h history of pollakiuria, dysuria and a painful abdomen on clinical examination. Lateral abdominal radiograph- and ultrasound examinations showed a large fluid containing structure with intraluminal hyperechoic masses, that was misinterpreted as the urinary bladder. A volume of 300 mL haemorrhagic fluid was drained by cystocentesis and, due to suspicion of a bladder malignancy, the case was referred for further evaluation.

On clinical examination the dog was bright and alert with a body condition score of 6/9, normal breathing, pulse 100/min, T 38.8 °C, and pink mucous membranes. The abdomen was painful on palpation. Haematology, biochemistry and coagulation tests (prothrombin time and partial thromboplastin time) were unremarkable apart from mild hyperkalaemia (5.3 mmol/L; reference range 3.4–4.9 mmol/L). To rule out a bleeding disorder, additional tests were performed: the buccal mucosal bleeding time was normal and a lungworm (*Angiostrongylus vasorum*) test was negative. Abdominal ultrasound revealed free echogenic fluid which was haemorrhagic on paracentesis with a packed cell volume (PCV) of 17% and total solids (TS) 32 g/L, compared to the peripheral blood which had a PCV of 48% and TS of 66 g/L. This excluded abdominal bleeding and raised suspicion of a cystic mass with occult bleeding within the cyst.

**Fig. 1:**
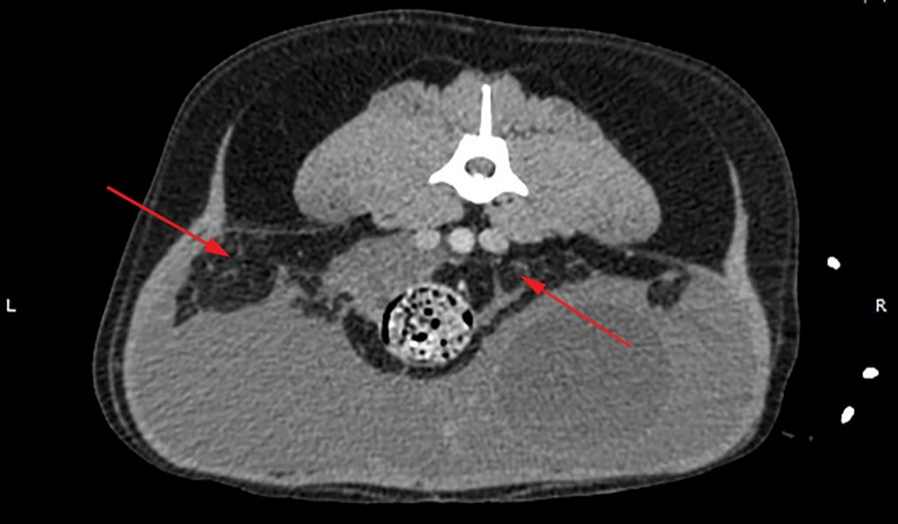
Transverse section of the abdominal cavity on CT-scan, without contrast. A large amount of free abdominal fluid is present. The arrows indicate the retroperitoneal space, which has a striated appearance, indicative of free fluid

**Fig. 2:**
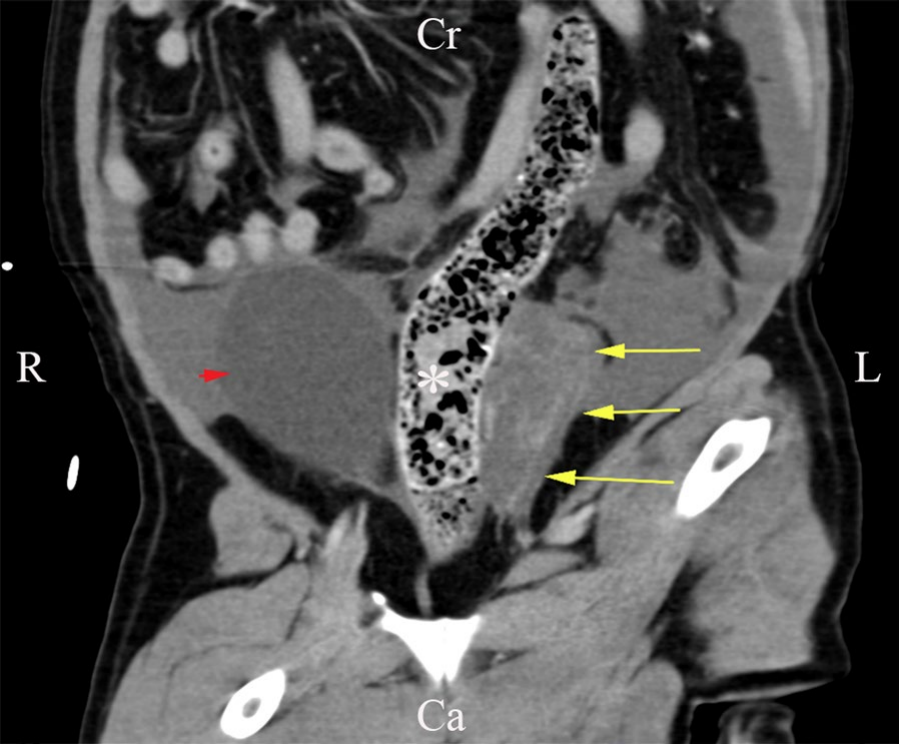
Dorsal section of the abdominal cavity on CT-scan, without contrast. The retroperitoneal cystic structure (arrow) is adjacent to the colon (asterisk). The urinary bladder (arrowhead) can be visualised on the right side of the colon. *R* right, *L* left, *Cr* cranial, *Ca* caudal

**Fig. 3:**
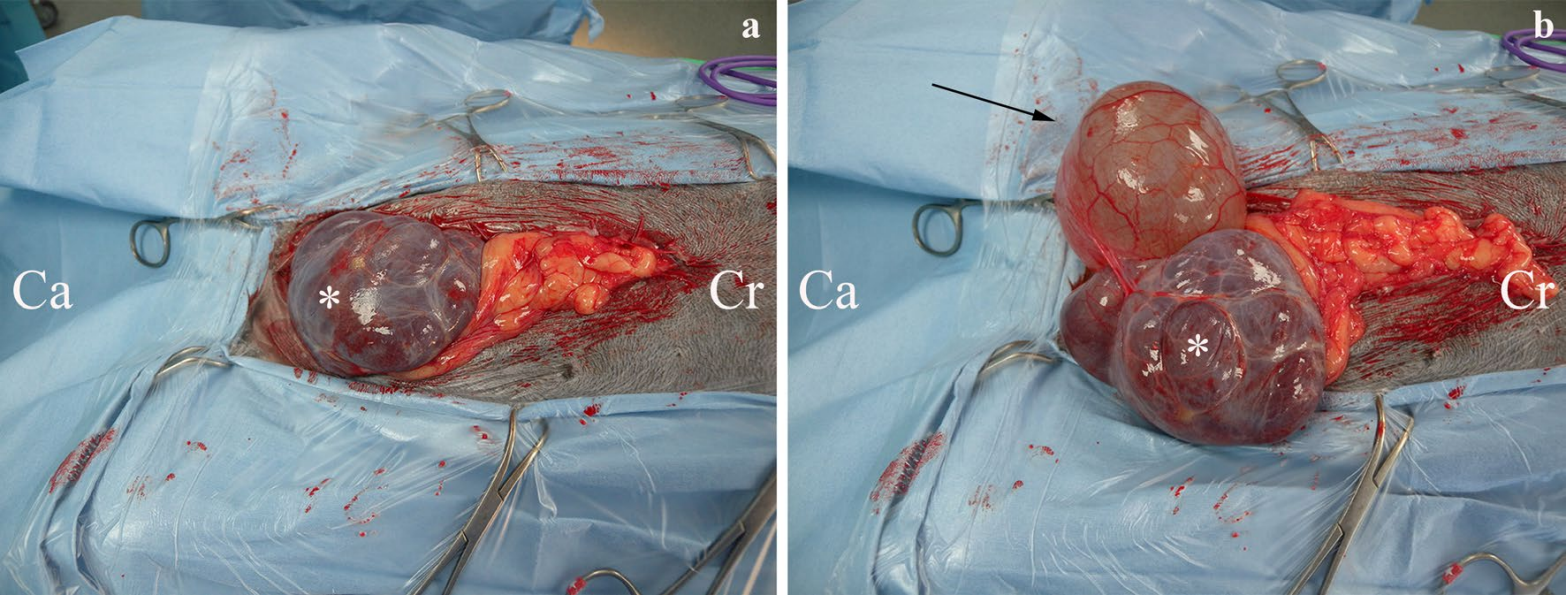
Intra-operative photographs of the retroperitoneal cystic structure and urinary bladder. **a** Shows the large cystic structure in the caudal abdomen, which could be misinterpreted as the bladder. **b** However, on thorough exploration of the abdomen, the bladder (arrow) can be identified on the right side of the patient. The cyst is marked with an asterisk (*). *Cr* cranial, *Ca* caudal

A thoracoabdominal computed tomography (CT) contrast-enhanced scan was performed. In the thoracic cavity mild atelectasis of no clinical significance was found, while in the abdomen a large volume of echogenic peritoneal fluid, with no contrast enhancement was found (Fig. 1). On the left side of the bladder, within pockets of ascites, a focal region of contrast-enhancing striated material was identified, adjacent to the descending colon, left femoral artery, uterine stump and left aspect of the bladder (Fig. 2). A single, small (2.5 mm diameter) soft tissue attenuating nodule was identified adjacent to the spleen, dorsally in the left side of the abdomen. The cystic structure was not aspirated again and the splenic nodule was too small to aspirate. Following these results, our differential diagnoses were neoplasia, fibrinous material or granulomatous inflammation. A ventral midline exploratory laparotomy from the xiphoid to the pubis was performed as a diagnostic and therapeutic measure. A large, thin-walled fluid-filled structure was found in the left side of the caudal abdomen, extending from the left retroperitoneal space, which had expanded the dorsal parietal peritoneum and caused it to be deviated to lie dorsal to the bladder. On the left side, the fluid had expanded within the left lateral bladder ligament and was surrounding the left ureter (Fig. 3). The uterine pedicle was not involved. The cystic structure was drained to distinguish pathologic cyst wall from normal anatomical structures. Both ureters were exposed, so they did not adhere to the cystic structure or the dorsal peritoneum. Most of the cyst wall (around 90%) was excised, while preserving the kidneys, ureters, bladder and internal and external iliac artery and vein. No additional cystic formations were identified in the retroperitoneal space, and there was no evidence of metastasis to the abdominal lymph nodes or organs. The splenic nodule seen on abdominal ultrasound could not be visualised macroscopically. The retroperitoneal space was omentalised to aid in ongoing drainage. The excised tissue was submitted for histological examination. The dog made an unremarkable recovery and was discharged 2 days post-operatively.

**Fig. 4:**
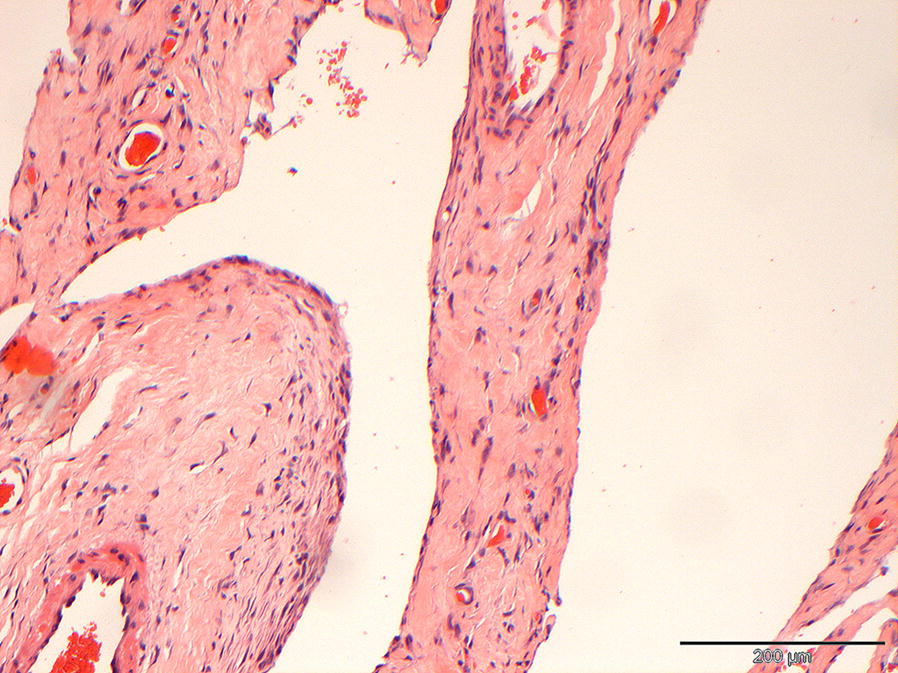
Histopathology of the cyst wall. The cyst wall is composed of dense fibrous tissue containing multiple well developed blood vessels. The cyst is lined by bland very slender spindle cells. Haematoxylin and eosin

**Fig. 5:**
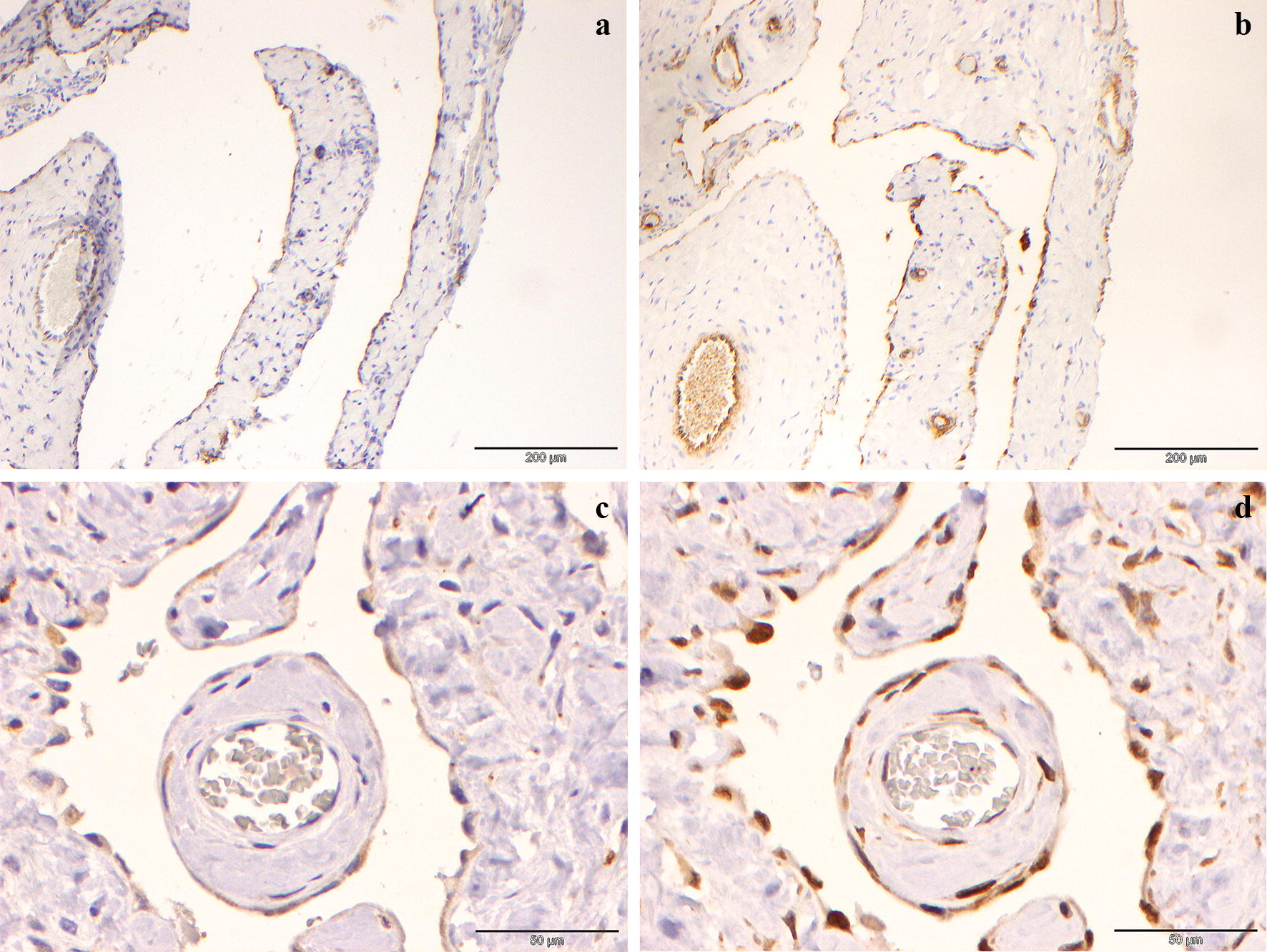
Immunostaining of the cyst wall. Both the spindle cells lining the cyst and those lining the mural blood vessels exhibit positive (brown) intracytoplasmic staining for CD31 (**a**) and von Willebrand Factor (**b**). This indicates a vascular origin of the cyst. **c** LYVE-1 staining of the cells lining the cyst wall is positive and varies from weak to moderate intracytoplasmic staining (brown). Note that the endothelial cells lining the blood vessel in the central papillary projection of cyst wall are negative. **d** PROX-1 staining of the cells lining the cyst wall is positive showing moderate to strong intranuclear staining (brown). Note the endothelial cells lining the blood vessel in the central papillary projection of cyst wall are negative

Histological examination of the excised tissue confirmed a cystic lesion composed of a fibrovascular capsule, with an inner layer of bland, flattened spindle cells (Fig. 4). The blood vessel density of the wall varied, but the vessels were well differentiated with occasional larger arteries with an expanded tunica media. Moderate to severe congestion was frequent, and multifocal mild to moderate perivascular haemorrhage was present. Small foci of haemosiderin-laden macrophages were observed in association with perivascular haemorrhage. Based on the very bland nature of the lining cells, the solitary nature of the lesion and lack of evidence of metastasis, malignancy was excluded. At this stage differential diagnoses included a cystic vascular lesion and, less likely, a cystic mesothelial proliferation. Immunohistochemistry was performed to differentiate between these conditions. The lining cells showed moderate positive intracytoplasmic staining for platelet endothelial cell adhesion molecule-1 (CD31) (Fig. 5a) and strong intracytoplasmic positive staining for von Willebrand factor (vWF) (Fig. 5b) and vimentin. No positive staining for cytokeratin was noticed (anti-acidic cytokeratin antibody-1 (AE-1)/AE3). The positive CD31 and vWF staining confirmed the suspicion of a vascular lesion. Given the almost absence of blood in the cyst by histology the suspicion of a lymphatic origin remained. Due to lack of commercial availability of lymphatic-specific staining, further differentiation was not possible at this moment.

Four months after surgery, an abdominal ultrasound examination revealed a localized multiloculated cystic structure in the same region where the initial cyst had been seen. This cystic structure was approximately half the size of the initial cyst. As the amount of fluid in the cystic lesion was small and the dog was without clinical signs, conservative treatment was opted and a re-check in 3 months’ time was advised.

Eleven months after the initial presentation, the dog was presented again with recurrence of a distended abdomen. Abdominal ultrasound revealed progression of the retroperitoneal lesion with a dilated ureter and a moderate hydronephrosis on the left side. Surgical exploration showed recurrence of a large single lumen cystic mass in the left retroperitoneal region, with multiple adhesions to bladder, loops of small intestine and terminal branches of the aorta. Hence, it was not possible to deroof the mass and partially excise the wall as it was the first time. The mass was dissected from the left ureter to relieve the obstruction and the cyst was drained. To be able to provide an entrance port for drainage, an implantable drainage system (PleuralPort; Norfolk Animal products, Skokie, IL, USA) was placed. The fenestrated part was placed in the cystic cavity without suturing and the injection port was placed in the left inguinal region. Follow-up consultations were scheduled with 3 months’ interval, to carry out ultrasound guided drainage of the cyst via the PleuralPort.

The lesion showed similar histomorphology to the initially excised mass. Immunohistochemistry for lymphatic markers (LYVE-1 and PROX-1) was performed and revealed weak to mild positive intracytoplasmic labelling for LYVE-1 (Fig. 5c) and moderate to strong intranuclear staining for PROX-1 (Fig. 5d), indicating a lymphatic origin to the cystic mass.

Fifteen months after initial presentation the dog was brought in for routine follow-up. Abdominal ultrasound revealed similar findings to the previous visit, with no further progression. No fluid could be drained from the PleuralPort. As the condition was stable, a follow-up in 3 months’ time was advised. Four days after this routine follow-up, the dog was presented for lethargy and weakness, with a PCV of 14% and TS of 41 g/L. Ultrasound of the abdomen revealed fluid accumulation in the cystic cavity, pleural- and abdominal spaces. Abdominocentesis was performed and haemorrhagic fluid was aspirated with a PCV of 21%. Coagulation tests was within normal limits. The owners did not wish to proceed with further procedures and opted for euthanasia. Post-mortem examination was declined by the owners.

## Discussion and conclusions

Lymphatic malformations in humans tend to occur in infants and are believed to arise from a congenital failure of primitive lymphatic sacs to develop communications with the venous system [2]. Mutations have been suggested as an underlying cause for lymphatic malformations in humans [25].

Only few case-reports about lymphatic malformations have been published in veterinary literature and classification of vascular malformations in veterinary medicine has not been well established due to their infrequency [16–21]. Gross et al. [25] classified lymphatic anomalies in dogs and cats and refers to lymphangiomatosis when the anomaly is caused by malformation, while lymphangioma has been suggested for a neoplastic origin. Whereas, in human medicine, lymphangiomatosis referrers to diffuse lymphatic malformations involving multiple organs [2]. As this scatters confusion, the authors suggest the use of the human nomenclature from the International Society for the Study of Vascular Anomalies, where non-neoplastic lymphatic lesions with a presumed congenital origin are referred to as lymphatic malformations [3, 27, 29].

Lymphatic malformations in the retroperitoneum and abdominal viscera are a rare entity and account for 2% of all lymphatic malformations in humans [30]. Although lymphatic malformations can be subclassified as microcystic (diameter ≤ 5 mm), macrocystic (> 5 mm) or combined; retroperitoneal malformations are mostly macrocystic [2, 30–32], as seen in our patient. The most common presenting signs in humans with retroperitoneal cystic lymphatic malformations are abdominal pain and abdominal distension, but patients can also be presented with a palpable mass, back pain, anorexia, fever, nausea and diarrhoea [33–39]. Haemorrhage within the cystic space is common, indicating recent trauma or spontaneous intralesional bleeding [27, 32]; therefore symptoms can occur when patients become older due to increased size of the cystic space. Our patient was presented with a painful abdomen, as described in humans, but also with pollakiuria and dysuria. The latter symptoms are most likely the results of compression of the bladder and/or urethra. Our patient had no history of recent trauma. Bleeding into the cyst could be due to a minor traumatic event, unnoticed by the owner, but spontaneous intra-cystic bleeding seemed more likely.

Initial evaluation of cystic structures by ultrasonography, CT scanning or magnetic resonance imaging is advised, however definitive diagnosis can only be made through histopathology [27, 31]. CT-scan provided the best pre-operative information in our patient, as it gave a better understanding of the origin of the lesion and its association to surrounding organs, compared to abdominal ultrasound. Histopathology shows that vascular malformations are composed of a single layer of flattened-to-slightly hobnailed endothelial cells, rimmed by rare pericytes, with macrocystic lesions having thicker, irregular coats of smooth muscle and/or fibrous tissue and possibly valves [26]. As differentiation between blood vessel or lymphatic origin can be challenging on histopathology, immunoreactions for antigens as podoplanin (D2-40 antibody), PROX-1, LYVE-1 and vascular endothelial growth factor receptor-3 (VEGFR-3) help to make a final diagnosis [23–28]. Positive staining for CD31 and vWF, and absence of staining for cytokeratin in our patient indicate a vascular origin of the cyst and effectively rules out a mesothelial origin. CD31 and vWF have been used by others to make a diagnosis of vascular malformations in canine patients; however definitive diagnosis of lymphatic origin was based on presumptions or appearance on histopathology [16, 18–21]. Although PROX-1 is not a lymphatic specific marker, it helps in the differentiation between blood vessel and lymphatic origin as lymphatic endothelium use PROX-1 as transcription factor during development, whereas blood vessel endothelium does not. LYVE-1 is a cell surface receptor for the extracellular matrix glycosaminoglycan hyaluronan (HA) and the HA receptor is almost exclusively expressed on moderately to well-differentiated lymphatic vessel and absent in blood vessels [22]. Hence positive staining for LYVE-1 and PROX-1 confirmed the suspicion of a lymphatic malformation.

Invasive or infiltrative growth is commonly associated with malignant behaviour. However, vascular malformations have been reported to have a progressive behaviour and a tendency to expand into surrounding tissues [2, 25, 26]. Progressive angiomatosis is well known for its invasive behaviour, which is also described in previous cases of canine vascular- and lymphatic malformations [8, 9, 25]. Thus, differentiation between a tumor and a vascular malformation can be challenging.

In humans, the ideal treatment for symptomatic or large retroperitoneal cystic lymphatic malformations is surgical resection to achieve complete excision [33–39], although this is not always possible, leading to frequent recurrence [23–29]. Recurrence after apparently complete surgical excision (up to 30% in some reports) is not uncommon [1, 17, 18, 32]. Surgery involves the risk of damage to surrounding tissues, persistent chylous ascites and re-enlargement of residual lesions [31]. Dissection of the cyst in our patient had to be performed with precision, due to close proximity and enveloping vital structures as the ureter and terminal branches of the aorta and the caudal vena cava. Omentalisation has not been opted as therapy before in lymphatic vascular malformations in veterinary or human patients. However, the omentum has been proven to actively participate in the immune response and has the ability of absorbing fluid, therefore its use in abdominal and thoracic surgery is widely accepted [40]. Once recurrence occurred in our patient, a PleuralPort was placed to pursue occasional drainage of the cyst via the subcutaneous access port. In humans, closed suction drains are occasionally placed in large macrocystic lymphatic malformations, and can be combined with sclerosing therapy [31]. Unfortunately, our patient deteriorated acutely due to active spontaneous bleeding into the lesion, with fluid extravasation into the abdominal- and thoracic cavities. Due to the connection between the peritoneum and the thoracic cavity in between the diaphragm and the psoas muscles, the fluid most likely leaked from the peritoneum into the thoracic cavity, as described before [40]. As the patient quickly deteriorated, the owner opted for euthanasia, which was also the outcome in other veterinary cases due to infiltrative behaviour of the malformations, inability of surgical resection or risk of recurrence [16, 21].

Despite documentation of recurrence of the cystic structures in some cases, long term prognosis of lymphatic malformations in dogs is not well-established [16–21]. No human data is available for long term prognosis in retroperitoneal cystic lymphatic malformations [33–39].

Vascular malformations should be taken into consideration in a patient presented with a retroperitoneal cyst containing haemorrhagic fluid. Additional imaging, histopathology and LYVE-1 and PROX-1 immunohistochemistry can be used to diagnose lymphatic malformations.

## Data Availability

All data generated or analyzed during this study are included in this article.

## Abbreviations

AE-1: Anti-acidic cytokeratin antibody-1
CD31: Platelet endothelial cell adhesion molecule-1
CT: Computed tomography
LYVE-1: Lymphatic Vessel Endothelial Hyaluronic Acid Receptor-1
PCV: Packed cell volume
PROX-1: Prospero Homeobox Protein-1
TS: Total solids
VEGFR-3: Vascular endothelial growth factor receptor-3
vWF: Von Willebrand factor
WHO: World Health Organization
